# Immune responses in fatalities involving opioids

**DOI:** 10.1080/20961790.2018.1558503

**Published:** 2019-01-17

**Authors:** Henry J. Carson

**Affiliations:** Linn County Medical Examiner’s Office, Cedar Rapids, IA, USA

**Keywords:** Forensic medicine, allergy, drug, analgesics, opioid, asthma, drug overdose, tryptases

## Abstract

In some cases of fatalities involving opioid use, the concentrations of detected opioids are not in the toxic range. Immune reactions can be triggered by opioid use, suggesting that immune response may be a factor in these cases. Autopsy cases from 2002–2012 were reviewed. Persons with physical, microscopic or serum evidence of allergic reactions and opioid use at autopsy were compared to persons who used opioids but had no such signs. Overall, 49 persons were identified who had used opioids, of which five had evidence of immune response. A medical history of asthma was significantly more common in persons with signs of immune response (*P* = 0.0244) and fatality (*P* = 0.0085) compared to normals. A history of asthma is suggestive of susceptibility to immunologic reactions to opioids, and correlates strongly with the cause of death.

## Introduction

Death from abuse of prescription or illicit opioids is acutely on the rise in the US [1]. Death from drug abuse is usually determined by toxicological examination of the blood or other body fluids in conjunction with an autopsy, scene investigation and detailed decedent history [2].

However, in some cases the concentrations of opioids present are not detected at toxic concentrations, let alone fatal concentrations [3]. Moreover, evidence of metabolites of the native drugs in these cases can demonstrate that several hours of survival took place after drug use. Thus, the mechanism of death may be unknown, even if the cause and manner of death are clear. Immune mediation may be a contributing factor of death in these cases, since immune reactions can be triggered by opioid use [3]. Opioids are known to stimulate mast cells, initiating the release of histamine and other vasoactive substances [4–6].

However, it is not known how often immune activation takes place with opioid use, or if a risk group can be identified. This study investigates what kinds of decedents who use opioids demonstrate allergic activity at the time of death, and whether there are identifiable factors that can be significantly associated with fatality and possibly predictive of greater risk.

## Materials and methods

Cases from a 10-year period (2002–2012) were reviewed. Persons with opioids detected by post-mortem toxicology were selected for evaluation. Two groups were generated from this population: persons with physical, microscopic or serum evidence of allergic or anaphylactic reactions; and persons who used opioids but had no such signs. The findings that could enter someone into the study group were effects such as a diffuse rash consistent with systemic mast cell degranulation; scratches consistent with acute pruritic symptoms from histamine release; systemic oedema suggestive of activity by chemical mediators released after IgE activation; gross lung findings such as mucus plugging of the bronchi, or microscopic evidence of lung infiltration by eosinophils with fibromuscular hyperplasia of the bronchioles, both indicative of asthma; or β mast-cell tryptase concentrations of serum measuring greater than 11.5 ng/mL, consistent with activation of mast cells in an allergic reaction. Other features of allergic reactions, such as pulmonary oedema, were not included, since they could be agonal changes, or otherwise caused by events not related to immune reaction. Data regarding demographics, clinical findings, toxicological studies, cause of death and manner of death were compared between the two groups. Statistical significance was tested using *t*-tests and *Χ*^2^ 2 × 2 contingency tables.

## Results

Demographic findings and clinical histories are summarized in Table 1. Overall, 49 persons were collected who had opioids identified at autopsy. Of these, five persons had evidence of immune system response, while 44 had no such factors identified. The factors that were noted included external scratches consistent with active pruritus (*n* = 2), gross and/or microscopic findings of asthma (*n* = 3), and elevated β mast-cell tryptase concentrations (*n* = 1; β mast-cell tryptase concentration 27.3 ng/mL). Neither rashes without scratches were observed, nor was diffuse oedema identified.

**Table 1.** Demographic findings and clinical histories (*N* = 49).

Items	Immune responders (*n* = 5)	Non-responders (*n* = 44)	*P*-value
Demographic findings			
Sex			
Male	5	23	NS
Female	0	21	
Race			
White	5	42	NS
Non-white	0	2	
Age (years)			
Mean	32	44	NS
Range	22–45	19–83	
Clinical histories			
Drug abuse	2	16	NS
Depression	3	12	NS
ASCVD	1	8	NS
Pneumonia	0	7	NS
Fatty liver	0	6	NS
Cirrhosis	0	5	NS
Emphysema	0	4	NS
Seizure disorder	0	4	NS
Obesity	0	2	NS
NIDDM	0	3	NS
Asthma	2	1	0.0244
Hypertension	1	1	NS

NS: not significant; ASCVD: atherosclerotic cardiovascular disease; NIDDM: non-insulin dependent diabetes mellitus.

Only one medical history was associated with persons who demonstrated immune response at autopsy, asthma (responders: *n* = 2, non-responders: *n* = 1; *P* = 0.0244). The drugs that were present in the cases are listed in Table 2. All persons had at least one opioid or metabolites identified. Either specific drug or combination of drugs were more likely to be associated with immune responders compared to non-responders. Cause and manner of death are summarized in Table 3. One cause of death, asthma, was significantly (*P* = 0.0085) more associated with responders (*n* = 2) compared to non-responders (*n* = 0). As regards manner of death, no significant associations were noted between immune responders and non-responders.

**Table 2.** Drugs found at autopsy (*N* = 49).

Drugs	Immune responders (*n* = 5)	Non-responders (*n* = 44)	*P*-value
Methadone	1	13	NS
Hydrocodone	1	10	NS
Oxycodone	1	9	NS
Morphine	0	8	NS
Heroin	2	4	NS
Hydromophone	0	3	NS
Codeine	0	2	NS
Fentanyl	0	1	NS
Benzodiazepine	3	15	NS
Zolpidem	0	2	NS
SSRI	1	17	NS
TCA	1	3	NS
Cyclobenzaprine	0	2	NS
Diphenhydramine	1	5	NS
β blocker	1	2	NS
Cocaine	0	6	NS
Amphetamine	0	1	NS
Pseudoephedrine	0	2	NS
Acetaminophen	1	6	NS
Propoxyphene	0	3	NS
Alcohol	0	8	NS
THC	1	7	NS

NS: not significant; SSRI: selective serotonin reuptake inhibitor; TCA: tricyclic antidepressant; THC: tetrahydrocannabinol.

**Table 3.** Cause and manner of death (*N* = 49).

Items	Immune responders (*n* = 5)	Non-responders (*n* = 44)	*P*-value
Cause of death			
MDI	2	27	NS
Pneumonia	0	5	NS
Overdose	0	5	NS
MVA	0	2	NS
PE	0	2	NS
GSW	0	2	NS
Asthma	2	0	0.0085
Leukemia	0	1	NS
Edema	1	0	NS
Manner of death			
Accident	4	32	NS
Natural	1	7	NS
Suicide	0	4	NS
Homicide	0	0	NS
Indeterminate	0	1	NS

MDI: multiple drug intoxication; NS: not significant; MVA: motor vehicle accident; PE: pulmonary embolus; GSW: gunshot wound.

## Discussion

Opioid use is a problem on the rise in the US. While prescription narcotic abuse is increasing, heroin is still common [1], and designer fentanyls are appearing in many areas [7]. Most fatalities from opioids are due to overdose, but deaths involving opioids with concentrations lower than those associated with fatality are common [3]. In this latter group with apparently sublethal concentrations of opioids, it can be difficult to interpret what the detected concentrations of opioids mean; pertinent factors that may be unanswerable even with comprehensive review of clinical history and medical records include individual tolerance of opioids or relative naïveté to the drugs; metabolism of the opioids if death is prolonged and the drugs remain as active metabolites; drug-drug interactions; and individual genetic factors, such as slow metabolizers. This study analyzed whether immune activation could also be a factor in fatality from opioids, based on findings in the autopsy that can be attributed to immune activation. While many signs of immune activation were observed, only those fatalities from asthma could be associated with opioid use at a statistically significant level (*P* = 0.0085).

There are many different phenotypes of asthma, including a near-fatal type, in which exposure to an activating substance or allergen, including heroin, can lead to rapid deterioration and death [8]. Part of the insidious nature of this reaction is that near-fatal asthma can impair antemortem perception of dyspnoea [8]. Heroin insufflation is a common exacerbation of native asthma [9], but cannot be considered the only dangerous route of heroin administration for asthmatics, since the heroin allergen is systemically distributed by injection as well [4,5]. Pinprick challenges with opiates have elicited allergic and asthmatic reaction in test subjects, although this method does not appear to be reliable for predicting which patients will respond to the challenge and which will not [10]. Another example of non-nasal heroin administration that included allergen reaction was an intrathecal administration that induced an allergic reaction, confirmed by β mast-cell tryptase concentrations and subsequent pinprick testing [11]. A large study of opioid addicts noted that 5% of them had a history of asthma, and that 1.4% of cases experienced the onset of asthmatic attacks after heroin use [12].

On initial or repeat exposure, a sensitized patient can release these vasoactive factors, possibly as an anaphylactoid reaction. This outcome appears to be what befell two subjects, who had asthmatic changes with low opioid concentrations. The reaction appears to be directly due to the effect of heroin on mast cells, and is independent of IgE concentrations or activity [13].

The use of postmortem β mast-cell tryptase to assess anaphylactic reactions is somewhat controversial. The concentration that denotes elevation due to allergic reaction is not clear; a recent study suggests that 53.8 ng/mL may be an appropriate cut-off [14]. A significant mean baseline elevation of β mast-cell tryptase has been noted in opioid users, however (6.0 ± 4.3 ng/mL), compared to non-opioid users (3.9 ± 1.9 ng/mL), suggesting that the standard threshold used in this study was appropriate [3]. Some studies have furthermore shown that this analyte may be elevated in postmortem serum of persons from whom anaphylactic reactions are not the cause of death [15,16]. It is important to use this test prudently, since increases of β mast-cell tryptase may be found incidentally. If the clinical or forensic setting of the death is consistent with an elevated β mast-cell tryptase concentration, however, it may be a useful adjunct to establishing the cause of death.

In summary, a history of asthma is suggestive of susceptibility to immunologic reactions and toxicity to opioids, and correlates strongly (*P* = 0.0085) with cause of death at autopsy. Testing for β mast-cell tryptase in any case of suspected opioid use may be valuable. When possible, it may be useful to save an aliquot of immediate-spin serum in a freezer in case subsequent β mast-cell tryptase testing would be indicated. While elevated β mast-cell tryptase results are not categorical evidence of an immunologic mechanism of death in the presence of opioids, it is a useful indictor in the constellation of scene investigation, clinical history and toxicological findings [2].

## Compliance of ethical standards

No patient-specific identifiers utilized in this study or report.

## Presentation

Abstract and platform presentation of these data at American Association of Forensic Sciences conference, Seattle WA, February 23, 2018.

## Disclosure statement

No potential conflict of interest was reported by the authors.
